# Classroom engagement among undergraduate students at a private college in Qingdao, China: a multidimensional analysis

**DOI:** 10.3389/fpsyg.2026.1780402

**Published:** 2026-08-28

**Authors:** Zhiyuan Yang, Ya’nan Lan, Qunqun Liu, Qijing Chen, Yuping Sun

**Affiliations:** Qingdao Hengxing University of Science and Technology, Qingdao, Shandong, China

**Keywords:** classroom engagement, gender differences, measurement invariance, private higher education, second-order confirmatory factor analysis, year of study

## Abstract

**Introduction:**

This study examined classroom engagement among undergraduate students at a private undergraduate college in Qingdao, China. Classroom engagement was conceptualized in terms of skills, emotional, interaction, and performance engagement.

**Methods:**

A quantitative cross-sectional design was employed with 534 undergraduate students. Descriptive statistics, Pearson correlations, confirmatory factor analysis, multigroup CFA, Welch’s independent-samples *t*-tests, and Welch’s ANOVA with Games–Howell *post hoc* comparisons were conducted using SPSS and AMOS.

**Results:**

The findings indicated moderate levels of classroom engagement, with performance engagement recording the highest mean and emotional engagement the lowest. All four dimensions were significantly and positively correlated (*r* = 0.410–0.517, *p* < 0.001). The second-order CFA demonstrated that all dimensions contributed substantially to the higher-order construct, with standardized loadings ranging from 0.63 to 0.74; interaction engagement had the highest loading. Scalar measurement invariance was supported across gender and year-of-study groups. Male students reported significantly higher scores than female students across all four dimensions, with small-to-moderate effect sizes. Significant year-of-study differences were also found, with first-year students generally reporting the highest engagement and fourth-year students the lowest.

**Discussion:**

These findings support the multidimensional structure of classroom engagement, extend evidence from Chinese private higher education, and highlight the importance of strengthening emotional engagement and sustaining classroom engagement throughout undergraduate study.

## 1 Introduction

Improving the quality of higher education is a central policy priority in China, with national reforms emphasizing student development, teaching quality, and the modernization of the higher education system [Central Committee of the Communist Party of China [CCCPC] and State Council [SPC], 2010; General Office of the Central Committee of the Communist Party of China [GCCPC] and General Office of the State Council [GOSC], 2019]. Within this policy context, students’ classroom engagement is an important indicator of how they participate in and experience teaching and learning.

Private higher education constitutes an important part of the higher education system in Shandong Province. By the end of 2023, the province had 44 private higher education institutions; students in these institutions accounted for 27.59% of new enrollments and 18.97% of the total higher education student population in the province (China Institute of Educational Science Research, 2023; Ministry of Education of the People’s Republic of China, 2023; Wang, 2020). These figures underscore the contribution of private institutions to expanding access to higher education.

The expansion of private higher education has also increased attention to teaching quality and students’ learning experiences. Differences in institutional resources, student backgrounds, learning support, and instructional arrangements may shape how students participate in classroom activities. Regional evidence further indicates that delivery mode may affect participation patterns. So et al. (2025) found comparable satisfaction across online and face-to-face courses in Hong Kong higher education but different patterns of enrollment and completion. This finding suggests that similar overall evaluations of learning experiences do not necessarily reflect equivalent engagement profiles. Because classroom engagement is associated with teaching quality and student development, examining engagement in private undergraduate education may provide useful evidence for institutional improvement (Li and Xue, 2023; Thornberg et al., 2022).

Nevertheless, research on classroom engagement in China has more often examined public universities, primary and secondary schools, or higher education in general than private undergraduate colleges. Evidence is particularly limited regarding the multidimensional structure of engagement and its comparability across demographic groups in private higher education. To address this gap, the present study investigates the levels and interrelationships of skills, emotional, interaction, and performance engagement among undergraduate students at a private college in Qingdao. It also examines the contribution of each dimension to a higher-order classroom engagement construct and differences by gender and year of study. The research questions were as follows.

1. What is the current status of classroom engagement among undergraduate students in a private undergraduate college in Qingdao, China?
2. What is the correlation among the four dimensions of classroom engagement (skills, emotional, interaction, and performance) among undergraduate students in a private undergraduate college in Qingdao, China?
3. What is the relative contribution of each dimension to overall classroom engagement?
4. Do the four dimensions of classroom engagement differ according to gender and year of study?

## 2 Literature

Astin (1984) student involvement theory conceptualizes student involvement as the physical and psychological energy that students devote to their academic experiences. From this perspective, engagement is reflected in the effort, attention, and participation that students invest in learning. Finn and Zimmer (2012) similarly described student engagement as active involvement in educational activities, encompassing both observable participation and internal psychological involvement. These perspectives indicate that classroom engagement is not limited to attendance or visible participation but also includes learning strategies, emotional connection, interaction, and performance-related effort.

Drawing on the Student Course Engagement Questionnaire developed by Handelsman et al. (2005), the present study conceptualizes classroom engagement as a multidimensional construct comprising skills, emotional, interaction, and performance engagement. Skills engagement refers to students’ use of learning strategies and study-related behaviors. Emotional engagement concerns students’ interest in and perceived connection with course content. Interaction engagement reflects participation in classroom communication and interaction with teachers and peers. Performance engagement concerns students’ effort and orientation toward academic achievement. Although these dimensions represent distinct aspects of engagement, they are theoretically related and may jointly reflect a broader classroom engagement construct. Accordingly, the present study examines whether the four first-order dimensions load onto a second-order classroom engagement factor.

**FIGURE 1:**
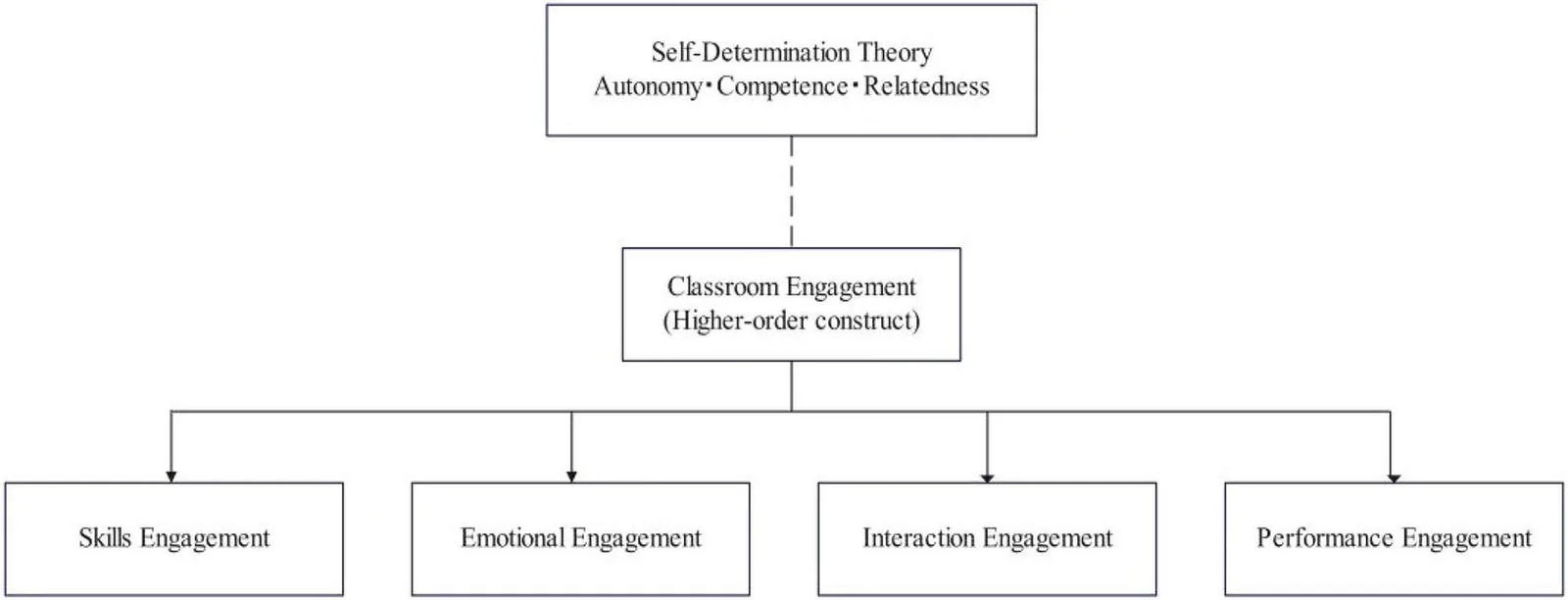
Conceptual framework of multidimensional classroom engagement.

Self-Determination Theory (SDT) provides an additional perspective for interpreting these dimensions. According to Ryan and Deci (2000), students are more likely to demonstrate sustained motivation and engagement when their needs for autonomy, competence, and relatedness are supported. Skills and performance engagement may be associated with students’ experiences of competence, whereas emotional and interaction engagement may be facilitated by feelings of connection and relatedness. Opportunities for active participation may also support autonomy. However, the four engagement dimensions are not treated as direct measures of the three psychological needs. Rather, SDT helps explain why supportive classroom conditions may be associated with students’ behavioral, emotional, and interactional involvement in learning. The conceptual framework is presented in Figure 1.

Classroom engagement has been associated with academic achievement, learning effectiveness, and the quality of students’ educational experiences (Mebert et al., 2020; Reeve and Lee, 2014). Research has also indicated that teacher support, autonomy-supportive instruction, learning motivation, and instructional design may be related to students’ engagement (Li and Xue, 2023; Núñez and León, 2019; Zainuddin et al., 2020). These findings suggest that engagement is shaped not only by students’ individual characteristics but also by their classroom and institutional environments.

Gender and year of study may be associated with classroom engagement, although these relationships are likely to depend on the educational context. For example, gender differences have been reported in the association between perceived teacher support and student engagement (Bru et al., 2021). Engagement may also vary across years of study as students encounter changing academic demands, course structures, internships, graduation requirements, and employment preparation. These context-dependent patterns highlight the need to examine gender and year-of-study differences in classroom engagement within private higher education.

Research on classroom engagement in China has covered primary and secondary schools as well as general higher education settings (Bai, 2016; Wang, 2022; Zhao et al., 2023). However, students in private undergraduate colleges remain comparatively underrepresented in this literature. Given the role of private institutions in expanding access to undergraduate education, examining classroom engagement in this context may extend current understanding of students’ learning experiences in Chinese higher education. Evidence is particularly limited regarding whether the four engagement dimensions form a higher-order construct and whether their measurement structure is comparable across gender and year-of-study groups in private undergraduate education.

Therefore, this study adopts the multidimensional framework proposed by Handelsman et al. (2005) to examine skills, emotional, interaction, and performance engagement among students at a private undergraduate college in Qingdao, China. It investigates the levels and interrelationships of the four dimensions, their contributions to a higher-order classroom engagement construct, and differences according to gender and year of study. In doing so, the study seeks to provide context-specific evidence concerning the structure and patterns of classroom engagement in Chinese private higher education.

## 3 Materials and methods

### 3.1 Participants and data collection

This study employed a cross-sectional questionnaire survey to investigate classroom engagement among undergraduate students at a private undergraduate college in Qingdao, China. A year-stratified convenience recruitment approach was used to obtain participation from students across all 4 years of undergraduate study. The questionnaire link was distributed to each year group through course instructors and class advisors using online communication platforms. Participation was voluntary, and no monetary or nonmonetary incentives were provided. All items were mandatory, and the online platform prevented the submission of incomplete questionnaires. Data were collected between November and December 2024. Of the 586 submitted questionnaires, 52 were excluded because of invalid response patterns, including invariant responding or completion in less than 50 s. The final sample comprised 534 valid responses (retention rate = 91.1%). The sample included students from all 4 years of study and was adequate for the CFA and group comparisons (Hair et al., 2019; Kline, 2016). Detailed demographic characteristics are presented in Table 1.

**TABLE 1:** Demographic characteristics.

Demographic variables	Categories	Frequency	Percentage (%)
Gender	Male	259	48.5
	Female	275	51.5
Year of study	Year 1	101	18.9
	Year 2	157	29.4
	Year 3	158	29.6
	Year 4	118	22.1

### 3.2 Instrumentation

The questionnaire used in this study was adapted from the Student Course Engagement Questionnaire (SCEQ) developed by Handelsman et al. (2005). The original SCEQ contains 23 items measuring four dimensions of classroom engagement. Following adaptation, the instrument used in this study comprised 17 items: seven measuring skills engagement, three measuring emotional engagement, four measuring interaction engagement, and three measuring performance engagement.

To ensure the suitability of the instrument for the Chinese private higher education context, a systematic adaptation process was undertaken. First, two experts in educational management reviewed the original items for conceptual relevance, clarity, and contextual appropriateness. Based on their recommendations, negatively worded, repetitive, or contextually less relevant items were revised or removed. The retained items were subsequently reviewed again by the same experts to evaluate their coverage of the four engagement dimensions.

A translation and back-translation procedure based on Brislin (1970) was then employed. Two bilingual Chinese–English researchers independently translated the retained items into Chinese and reconciled their translations through discussion. A separate bilingual expert who had not participated in the initial translation subsequently back-translated the Chinese version into English. Differences between the original and back-translated versions were discussed and resolved to ensure semantic equivalence. The translated Chinese version was subsequently reviewed by two experts in Chinese language education to improve its linguistic clarity and cultural appropriateness.

Before formal data collection, the adapted questionnaire was pilot-tested with 71 undergraduate students who were not included in the main sample. The pilot study evaluated comprehensibility, internal consistency, and item discrimination. As shown in Table 2, Cronbach’s alpha coefficients ranged from 0.829 to 0.906, and corrected item–total correlations ranged from 0.383 to 0.625. All corrected item–total correlations exceeded 0.30, indicating acceptable item discrimination. These results supported the use of the adapted instrument in the main study.

**TABLE 2:** Pilot study reliability and item analysis results (*N* = 71).

Dimension	Number of items	CITC rang	Cronbach’s α
Skills engagement	7	0.400–0.625	0.906
Emotional engagement	3	0.383–0.488	0.829
Interaction engagement	4	0.388–0.436	0.900
Performance engagement	3	0.428–0.611	0.868

All items were rated on a five-point Likert scale ranging from 1 (strongly disagree) to 5 (strongly agree). Scores for each dimension were calculated by averaging the corresponding items, with higher scores indicating higher levels of engagement in that dimension. No observed overall composite score was used in the primary inferential analyses. Instead, overall classroom engagement was represented as a second-order latent construct in the CFA. The complete Chinese and English versions of the instrument are provided in Appendix 1.

### 3.3 Data analysis

Data were analyzed using IBM SPSS Statistics 29.0 and AMOS 27.0. Descriptive statistics were calculated for the four classroom engagement dimensions, and distributional properties were assessed using skewness and kurtosis. Because the dimension scores were derived from five-point Likert items, they were treated as approximately continuous. Pearson correlations with 95% confidence intervals were used to examine relationships among the four dimensions. No item-level data were missing because the online questionnaire required a response to every item.

Cronbach’s alpha, McDonald’s omega, composite reliability (CR), and average variance extracted (AVE) were used to evaluate reliability and convergent validity. Confirmatory factor analysis (CFA) with maximum likelihood estimation was conducted to assess the four-factor measurement structure and the second-order classroom engagement model. Model fit was evaluated using χ^2^/df, IFI, TLI, CFI, and RMSEA. The second-order latent-variable model was used instead of an observed overall score calculated from the same items.

Multigroup CFA was conducted to test configural, metric, and scalar invariance across gender and year-of-study groups. Measurement invariance was considered supported when |ΔCFI| ≤ 0.010 and the increase in RMSEA was ≤ 0.015. Following the establishment of scalar invariance, gender differences were examined using Welch’s independent-samples *t*-tests, with Cohen’s *d* reported as the effect-size measure. Year-of-study differences were examined using Welch’s one-way ANOVA followed by Games–Howell *post hoc* comparisons, with η^2^ reported as the effect-size measure. All inferential tests were two-tailed, with statistical significance set at *p* < 0.05.

## 4 Results and discussion

### 4.1 Demographics

The final sample consisted of 534 undergraduate students from a private undergraduate college in Qingdao, China. The demographic characteristics of the participants are presented in Table 1.

As shown in Table 1, the sample included 259 male students (48.5%) and 275 female students (51.5%), indicating a relatively even gender distribution. Third-year students constituted the largest group (29.6%), closely followed by second-year students (29.4%), fourth-year students (22.1%), and first-year students (18.9%). All 4 years of study were represented, providing sufficient group coverage for the subsequent gender and year-of-study analyses.

### 4.2 Measurement model assessment

A second-order confirmatory factor analysis (CFA) was conducted to assess the construct validity of the classroom engagement scale and determine whether its four dimensions represented a higher-order construct. In this model, the 17 observed items were specified as indicators of four first-order factors—skills engagement, emotional engagement, interaction engagement, and performance engagement—which were subsequently specified as indicators of the second-order classroom engagement construct. Model adequacy was evaluated using multiple goodness-of-fit indices, including the chi-square-to-degrees-of-freedom ratio (CMIN/DF), comparative fit index (CFI), Tucker–Lewis index (TLI), incremental fit index (IFI), and root mean square error of approximation (RMSEA). The results are presented in Table 3.

**TABLE 3:** Goodness-of-fit indices for the second-order CFA model.

Type of fit	Fit measure	Index	Criterion	Interpretation
Absolute fit	RMSEA	0.008	< 0.08	Excellent
Incremental fit	CFI	0.999	> 0.90	Excellent
	TLI	0.999	> 0.90	Excellent
	IFI	0.999	> 0.90	Excellent
Chi-square	CMIN/DF	1.037	< 3.00	Excellent

As shown in Table 3, the second-order CFA model demonstrated an excellent fit to the data. The RMSEA was 0.008, which was well below the recommended threshold of 0.08. The incremental fit indices also exceeded the recommended criterion of 0.90, with CFI = 0.999, TLI = 0.999, and IFI = 0.999. In addition, the chi-square-to-degrees-of-freedom ratio (CMIN/DF) was 1.037, which was substantially below the recommended threshold of 3.00. Collectively, these results indicate that the proposed second-order CFA model adequately represented the observed data and provide strong support for the higher-order structure and construct validity of the classroom engagement scale.

**FIGURE 2:**
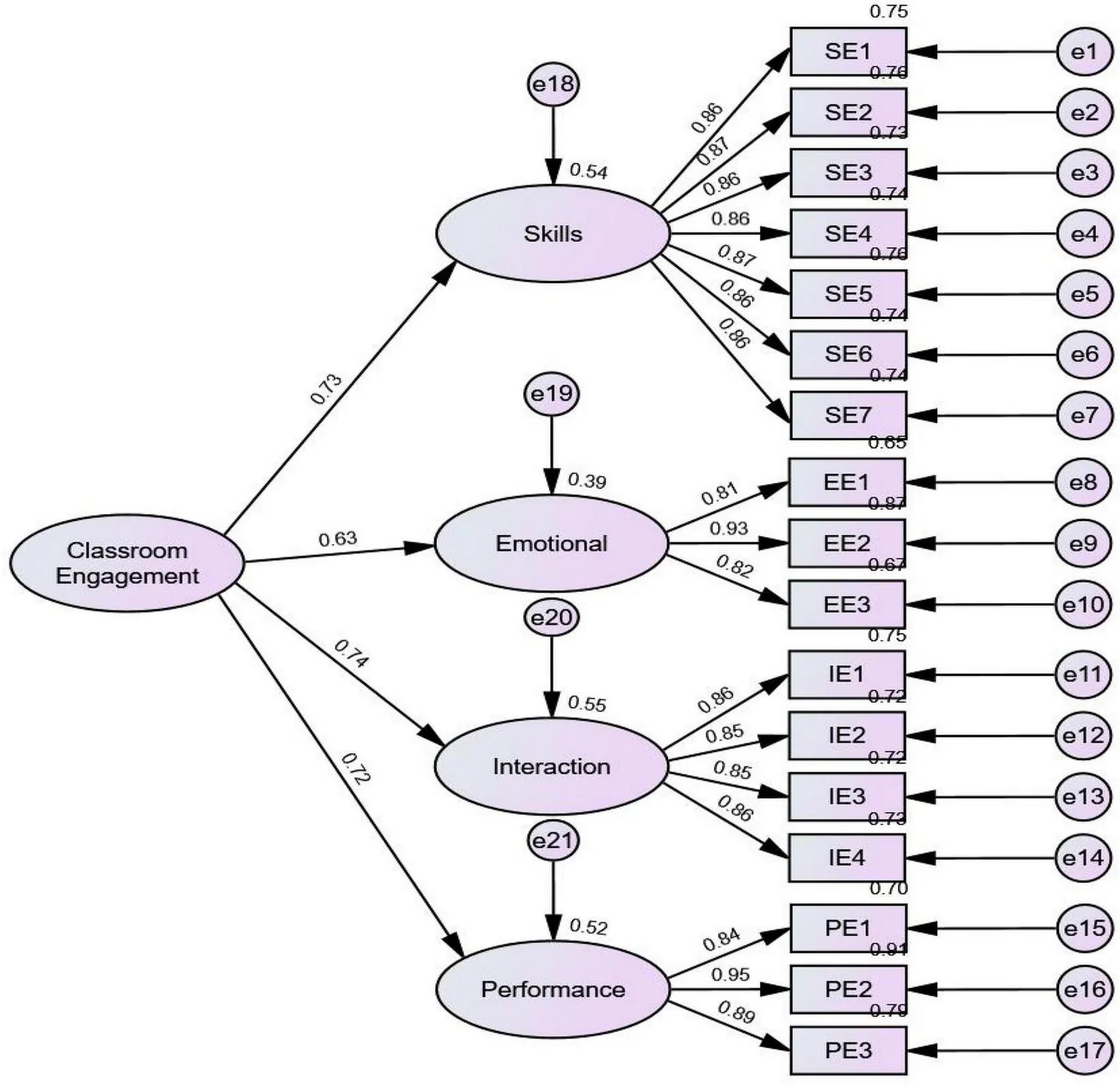
Second-order confirmatory factor analysis model of classroom engagement.

The standardized second-order CFA model is presented in Figure 2. The figure illustrates the relationships between the 17 observed items, the four first-order dimensions, and the higher-order classroom engagement construct.

As illustrated in Figure 2, all 17 items loaded on their corresponding first-order factors. At the second-order level, interaction engagement demonstrated the highest standardized loading on classroom engagement (β = 0.744), followed by skills engagement (β = 0.731), performance engagement (β = 0.721), and emotional engagement (β = 0.626). These results indicate that all four dimensions were meaningful indicators of the higher-order classroom engagement construct, although the strength of their associations with the higher-order construct varied. The exact standardized item loadings are reported in Table 4.

**TABLE 4:** Standardized factor loadings of the classroom engagement scale.

Dimension	Item	Standardized loading
Skills engagement	SE1	0.864
	SE2	0.873
	SE3	0.855
	SE4	0.857
	SE5	0.872
	SE6	0.861
	SE7	0.862
Emotional engagement	EE1	0.805
	EE2	0.934
	EE3	0.819
Interaction engagement	IE1	0.864
	IE2	0.849
	IE3	0.851
	IE4	0.855
Performance engagement	PE1	0.836
	PE2	0.953
	PE3	0.887

SE, skills engagement; EE, emotional engagement; IE, interaction engagement; PE, performance engagement.

To further evaluate the reliability and convergent validity of the measurement model, Cronbach’s alpha, McDonald’s omega, composite reliability (CR), and average variance extracted (AVE) were calculated for each first-order dimension. The results are presented in Table 5.

**TABLE 5:** Composite reliability and convergent validity of the measurement model.

Dimension	Cronbach’s α (95% CI)	McDonald’s ω	CR	AVE	SE	EE	IE	PE
SE	0.953 (0.949, 0.958)	0.954	0.954	0.746	–	–	–	–
EE	0.886 (0.871, 0.898)	0.890	0.890	0.730	0.485			
IE	0.916 (0.905,0.924)	0.916	0.916	0.731	0.534	0.456		
PE	0.919 (0.906, 0.930)	0.921	0.922	0.798	0.528	0.452	0.564	

SE, skills engagement; EE, emotional engagement; IE, interaction engagement; PE, performance engagement. Confidence intervals for Cronbach’s alpha were estimated using 5,000 bias-corrected and accelerated bootstrap resamples.

As presented in Table 5, Cronbach’s alpha coefficients ranged from 0.886 to 0.953, and the lower bounds of their 95% BCa bootstrap confidence intervals were all above 0.70. McDonald’s omega coefficients ranged from 0.890 to 0.954. All coefficients exceeded the conventional threshold of 0.70, indicating satisfactory internal consistency (Hair et al., 2019). The CR values ranged from 0.890 to 0.954, exceeding the recommended threshold of 0.70, whereas the AVE values ranged from 0.730 to 0.798, exceeding the recommended threshold of 0.50, thereby supporting construct reliability and convergent validity (Fornell and Larcker, 1981; Hair et al., 2019). In addition, the HTMT ratios ranged from 0.452 to 0.564 and were below the conservative threshold of 0.85, supporting discriminant validity (Henseler et al., 2015). Together with the standardized factor loadings reported in Table 4, these results provide evidence for the reliability, convergent validity, and discriminant validity of the four engagement dimensions.

### 4.3 Measurement invariance across gender and year of study

Before comparing classroom engagement scores across demographic groups, multigroup CFA was conducted to examine whether the four-factor measurement model operated equivalently across gender and year-of-study groups. Configural, metric, and scalar invariance were assessed sequentially. The model-fit indices for the invariance models are presented in Table 6, and the changes in fit between successive models are reported in Table 7.

**TABLE 6:** Measurement invariance model-fit indices across gender and year of study.

Grouping variable	Model	χ^2^	df	χ^2^/df	CFI	TLI	RMSEA
Panel A. gender	Configural	256.568	226	1.135	0.996	0.995	0.016
	Metric	271.413	239	1.136	0.996	0.995	0.016
	Scalar	283.663	252	1.126	0.996	0.995	0.015
Panel B. year of study	Configural	552.391	453	1.219	0.987	0.984	0.020
	Metric	614.631	492	1.249	0.984	0.982	0.022
	Scalar	653.947	531	1.232	0.983	0.983	0.021

**TABLE 7:** Changes in model fit across nested measurement invariance models.

Grouping variable	Model comparison	Δχ^2^	Δdf	*p*	ΔCFI	ΔRMSEA	Decision
Panel A. gender	Metric vs. configural	14.845	13	0.317	0.000	0.000	Supported
	Scalar vs. metric	12.250	13	0.507	0.000	−0.001	Supported
Panel B. year of study	Metric vs. configural	62.240	39	0.010	−0.003	0.002	Supported
	Scalar vs. metric	39.316	39	0.456	−0.001	−0.001	Supported

For gender, the configural model demonstrated an excellent fit to the data, χ^2^(226) = 256.568, χ^2^/df = 1.135, CFI = 0.996, TLI = 0.995, and RMSEA = 0.016. This result indicated that the same four-factor structure was supported for male and female students. Constraining the factor loadings to equality did not meaningfully reduce model fit, Δχ^2^(13) = 14.845, *p* = 0.317, ΔCFI = 0.000, and ΔRMSEA = 0.000, supporting metric invariance. The scalar model also produced no meaningful deterioration in fit relative to the metric model, Δχ^2^(13) = 12.250, *p* = 0.507, ΔCFI = 0.000, and ΔRMSEA = −0.001. Therefore, scalar invariance was established across gender.

The configural model across the 4 year-of-study groups also demonstrated a satisfactory fit, χ^2^(453) = 552.391, χ^2^/df = 1.219, CFI = 0.987, TLI = 0.984, and RMSEA = 0.020. During estimation, a small negative residual variance was identified for EE2 in the first-year group. Its residual variance was fixed at 0.004, and this specification was retained across all subsequent nested models. Following this adjustment, the remaining parameter estimates were within admissible ranges, and the conclusions regarding metric and scalar invariance remained substantively unchanged.

When the factor loadings were constrained to equality across the 4 year groups, the chi-square difference test was statistically significant, Δχ^2^(39) = 62.240, *p* = 0.010. However, the changes in the practical fit indices remained well within the recommended criteria, ΔCFI = −0.003 and ΔRMSEA = 0.002. Metric invariance was therefore considered supported. The scalar model did not differ significantly from the metric model, Δχ^2^(39) = 39.316, *p* = 0.456, and the changes in fit were negligible, ΔCFI = −0.001 and ΔRMSEA = −0.001. These findings supported scalar invariance across years of study.

Overall, the results indicated that the items measured the same underlying dimensions and had comparable measurement properties across gender and year-of-study groups. Establishing scalar invariance provided the necessary measurement basis for subsequently comparing the mean levels of skills, emotional, interaction, and performance engagement across these groups.

### 4.4 Descriptive statistics and normality assessment

Before conducting the main analyses, the distributional properties of the four classroom engagement dimensions were examined. As shown in Table 8, skewness values ranged from 0.053 to 0.207, whereas kurtosis values ranged from −1.523 to −1.266. All values were within the recommended range of ± 2 (Kline, 2016), indicating no substantial departure from normality. The distributional characteristics and sample size therefore supported the use of maximum likelihood estimation and parametric analyses.

**TABLE 8:** Descriptive statistics and normality assessment of classroom engagement dimensions.

Dimension	*N*	Minimum	Maximum	*M*	*SD*	Skewness	Kurtosis
SE	534	1.00	5.00	3.09	1.21	0.061	−1.523
EE	534	1.00	5.00	2.92	1.15	0.207	−1.351
IE	534	1.00	5.00	3.12	1.18	0.053	−1.390
PE	534	1.00	5.00	3.15	1.23	0.147	−1.266

SE, skills engagement; EE, emotional engagement; IE, interaction engagement; PE, performance engagement.

### 4.5 Classroom engagement among students in a private higher education institution

*RQ1*: What is the current status of classroom engagement among undergraduate students in a private undergraduate college in Qingdao, China?

As shown in Table 8, the four dimensions of classroom engagement recorded mean scores close to the midpoint of the five-point scale. Performance engagement had the highest mean score (*M* = 3.15, SD = 1.23), followed by interaction engagement (*M* = 3.12, SD = 1.18) and skills engagement (*M* = 3.09, SD = 1.21). Emotional engagement had the lowest mean score (*M* = 2.92, SD = 1.15). Overall, these findings indicate moderate levels of classroom engagement among the participating students, although emotional engagement was comparatively weaker than the other dimensions.

Skills engagement was moderate, indicating that students participated to some extent in activities involving note-taking, study strategies, and the application of learning skills. However, the dispersion of the scores suggests that students differed considerably in the extent to which they engaged in these activities. This finding supports the importance of providing structured opportunities for students to develop and apply effective learning skills. Consistent with Büchele (2021), skill-oriented learning activities may constitute an important component of students’ broader classroom engagement experiences.

Emotional engagement recorded the lowest mean among the four dimensions. Although the result does not indicate uniformly low emotional engagement, it suggests that students’ emotional connection with classroom learning was comparatively less developed than their skills, interaction, and performance engagement. This finding is important because students’ interest, enjoyment, and sense of connection with learning may influence their willingness to sustain classroom participation. It is also consistent with research emphasizing the role of emotional engagement in students’ motivation and sense of belonging (Collins et al., 2023).

Interaction engagement was the second-highest dimension, suggesting that students generally demonstrated moderate involvement in classroom communication and participatory activities. This result supports the view that interaction and participation constitute important components of classroom engagement (Zhao et al., 2023). Nevertheless, the moderate rather than high mean indicates that opportunities for student–teacher and peer interaction could still be strengthened.

Performance engagement had the highest mean score, indicating that students placed comparatively greater emphasis on classroom activities associated with academic performance and achievement. This finding is consistent with previous research demonstrating an association between student engagement and academic performance in higher education (Reeve and Lee, 2014). Nevertheless, the mean score remained within the moderate range, indicating that performance engagement was comparatively higher than the other dimensions but not high in absolute terms.

Taken together, the findings demonstrate that classroom engagement in the sampled private undergraduate college was moderate and multidimensional. Students showed comparatively stronger performance and interaction engagement but relatively weaker emotional engagement. This pattern suggests that students may be more attentive to observable participation and academic outcomes than to their emotional connection with classroom learning. Accordingly, efforts to improve classroom engagement should address not only students’ learning behaviors and performance expectations but also their interest, enjoyment, and sense of connection with classroom activities.

*RQ2*: What is the correlation among the four dimensions of classroom engagement (skills, emotional, interaction, and performance) among undergraduate students in a private undergraduate college in Qingdao, China?

Table 9 presents the Pearson correlations among the four dimensions of classroom engagement, together with their 95% confidence intervals. All dimensions were significantly and positively correlated with one another, with correlation coefficients ranging from *r* = 0.410 to *r* = 0.517 (*p* < 0.001). The confidence intervals for all correlations excluded zero, providing further evidence that the observed associations were statistically significant.

**TABLE 9:** Correlations among the four dimensions of classroom engagement.

Relationship	*r*	95% CI	*p*
Skills–emotional	0.449	(0.379, 0.514)	< 0.001
Skills–interaction	0.499	(0.432, 0.560)	< 0.001
Skills–performance	0.495	(0.428, 0.556)	< 0.001
Emotional–interaction	0.413	(0.340, 0.481)	< 0.001
Emotional–performance	0.410	(0.337, 0.479)	< 0.001
Interaction–performance	0.517	(0.452, 0.577)	< 0.001

The strongest correlation was observed between interaction engagement and performance engagement, *r* = 0.517, 95% CI (0.452, 0.577), *p* < 0.001. This was followed by the correlations between skills engagement and interaction engagement, *r* = 0.499, 95% CI (0.432, 0.560), *p* < 0.001, and between skills engagement and performance engagement, *r* = 0.495, 95% CI (0.428, 0.556), *p* < 0.001. The weakest correlation was found between emotional engagement and performance engagement, *r* = 0.410, 95% CI (0.337, 0.479), *p* < 0.001. Nevertheless, this association remained moderate and statistically significant.

These findings indicate that students who reported higher engagement in one dimension also tended to report higher engagement in the other dimensions. However, the moderate magnitudes of the correlations suggest that the four dimensions were related but not interchangeable, with each capturing a distinct aspect of classroom engagement. Interaction engagement showed comparatively stronger associations with skills and performance engagement. In the context of the participating private college, students’ classroom communication, question asking, group participation, and peer assistance may be closely associated with their use of learning strategies and attention to academic performance. This pattern is consistent with the multidimensional framework proposed by Handelsman et al. (2005). When considered together with the second-order CFA results, the findings support the conceptualization of classroom engagement as a higher-order construct comprising four related but distinguishable dimensions.

*RQ3*: What is the relative contribution of each dimension to overall classroom engagement?

Table 10 presents the contributions of the four dimensions to the higher-order construct of classroom engagement. All four dimensions loaded substantially on the second-order factor, with standardized loadings ranging from 0.63 to 0.74. Interaction engagement had the highest loading (β = 0.74, *R*^2^ = 0.55), followed by skills engagement (β = 0.73, *R*^2^ = 0.54), performance engagement (β = 0.72, *R*^2^ = 0.52), and emotional engagement (β = 0.63, *R*^2^ = 0.39).

**TABLE 10:** Relative contribution of the four dimensions to overall classroom engagement.

Dimension	Standardized loading (β)	*R* ^2^	Rank
Skills engagement	0.73	0.54	2
Emotional engagement	0.63	0.39	4
Interaction engagement	0.74	0.55	1
Performance engagement	0.72	0.52	3

The relatively high loading of interaction engagement indicates that, in the present sample, students’ communication and participation in classroom activities were particularly closely aligned with the broader construct of classroom engagement. Interaction provides observable opportunities for students to ask questions, communicate with teachers and peers, and participate actively in learning activities. This interpretation is consistent with Collins et al. (2023), who emphasized the importance of interaction and participation in students’ engagement experiences. However, the higher loading should not be interpreted as evidence that interaction engagement causes overall classroom engagement or is necessarily more important in every educational context.

Skills and performance engagement also had substantial loadings, indicating that students’ use of learning strategies and attention to academic performance represented important components of classroom engagement. Performance engagement recorded the highest mean but not the highest second-order loading. This distinction suggests that students generally placed comparatively greater emphasis on performance-related activities, whereas differences in interaction engagement were more closely aligned with differences in the higher-order engagement construct. In the context of private higher education, the relatively high performance mean may reflect students’ attention to academic achievement, graduation requirements, and future employment.

Emotional engagement had the lowest mean and the lowest second-order loading, although its loading remained substantial. One possible explanation is that students may participate in classroom tasks and pursue performance goals without developing an equally strong emotional connection with course content. From the perspective of Self-Determination Theory, students’ emotional involvement may depend partly on whether classroom experiences support their needs for autonomy, competence, and relatedness (Núñez and León, 2019). The comparatively lower emotional engagement observed in this study therefore highlights the importance of designing relevant learning activities, providing supportive feedback, and strengthening students’ sense of connection with classroom learning.

*RQ4*: Do the four dimensions of classroom engagement differ according to gender and year of study?

Before examining demographic differences, measurement invariance was assessed across gender and year-of-study groups. As reported in Section 4.3, scalar invariance was established, supporting meaningful comparisons of the four classroom engagement dimensions across these groups. The following sections present the gender and year-of-study comparison results.

*RQ4a*: Do the four dimensions of classroom engagement differ according to gender?

Gender differences in skills, emotional, interaction, and performance engagement were examined using Welch’s independent-samples *t*-tests. Cohen’s *d* and its 95% confidence interval were reported to indicate the magnitude and precision of the differences. The results are presented in Table 11.

**TABLE 11:** Gender differences in the dimensions of classroom engagement.

Dimension	Male *M* (*SD*)	Female *M* (*SD*)	Welch’s *t* (df)	*p*	Mean difference (95% CI)	Cohen’s *d* (95% CI)
SE	3.37 (1.18)	2.83 (1.18)	5.300 (530.271)	< 0.001	0.541 (0.341, 0.742)	0.459 (0.287, 0.631)
EE	3.19 (1.15)	2.68 (1.10)	5.260 (526.146)	< 0.001	0.511 (0.320, 0.703)	0.456 (0.284, 0.628)
IE	3.31 (1.15)	2.95 (1.19)	3.568 (531.466)	< 0.001	0.361 (0.162, 0.559)	0.309 (0.138, 0.479)
PE	3.47 (1.22)	2.86 (1.16)	5.910 (525.277)	< 0.001	0.609 (0.406, 0.811)	0.513 (0.340, 0.685)

SE, skills engagement; EE, emotional engagement; IE, interaction engagement; PE, performance engagement.

As shown in Table 11, statistically significant gender differences were found in all four dimensions of classroom engagement. Male students reported higher skills engagement (*M* = 3.37, SD = 1.18) than female students (*M* = 2.83, SD = 1.18), *t*(530.271) = 5.300, *p* < 0.001. The mean difference was 0.541, 95% CI (0.341, 0.742), and the effect size was small to moderate, *d* = 0.459, 95% CI (0.287, 0.631).

Male students also reported higher emotional engagement (*M* = 3.19, SD = 1.15) than female students (*M* = 2.68, SD = 1.10), *t*(526.146) = 5.260, *p* < 0.001. The mean difference was 0.511, 95% CI (0.320, 0.703), with a small-to-moderate effect size, *d* = 0.456, 95% CI (0.284, 0.628).

A statistically significant difference was also observed in interaction engagement. Male students (*M* = 3.31, SD = 1.15) reported higher scores than female students (*M* = 2.95, SD = 1.19), *t*(531.466) = 3.568, *p* < 0.001. The mean difference was 0.361, 95% CI (0.162, 0.559). The corresponding effect size was relatively small, *d* = 0.309, 95% CI (0.138, 0.479).

The largest gender difference was found in performance engagement. Male students (*M* = 3.47, SD = 1.22) reported higher performance engagement than female students (*M* = 2.86, SD = 1.16), *t*(525.277) = 5.910, *p* < 0.001. The mean difference was 0.609, 95% CI (0.406, 0.811), and the effect size was moderate, *d* = 0.513, 95% CI (0.340, 0.685).

Overall, in response to RQ4a, significant gender differences were observed across all four dimensions of classroom engagement in the present sample. The largest difference was found in performance engagement, followed by skills and emotional engagement, whereas the smallest difference was observed in interaction engagement. However, the effect sizes ranged from small to moderate, indicating that the practical magnitude of the observed gender differences was limited.

The comparatively larger difference in performance engagement may suggest that male students in this sample placed greater emphasis on performance-related classroom activities or perceived stronger connections between classroom participation and academic outcomes. The differences in skills and emotional engagement may also reflect variation in students’ perceived competence, classroom experiences, learning expectations, or responses to the instructional environment.

The observed differences should be interpreted as sample- and context-specific patterns rather than as stable gender-based characteristics. Such patterns may reflect differences in institutional context, academic discipline, instructional practices, and students’ learning experiences. Future research could examine the factors underlying these gender differences.

From a practical perspective, the findings suggest that institutions should examine whether male and female students experience classroom activities, participation opportunities, feedback, and academic support differently. Rather than adopting gender-stereotyped interventions, educators should provide inclusive forms of participation, varied learning activities, supportive feedback, and equitable opportunities for students to develop skills, interact with others, establish emotional connections with learning, and pursue academic performance goals.

*RQ4b*: Do the four dimensions of classroom engagement differ across years of study?

Differences in skills, emotional, interaction, and performance engagement across the 4 years of study were examined using Welch’s one-way ANOVA. Because the assumption of homogeneity of variance was not satisfied for all dimensions and the group sizes were unequal, Games–Howell *post hoc* comparisons were conducted following statistically significant omnibus tests. Eta-squared (η^2^) and its 95% confidence interval were reported as measures of effect size. The results are presented in Table 12.

**TABLE 12:** Differences in classroom engagement across years of study.

Dimension	Year 1 *M* (*SD*)	Year 2 *M* (*SD*)	Year 3 *M* (*SD*)	Year 4 *M* (*SD*)	Welch’s *F* (df1, df2)	*p*	η^2^ (95% CI)
SE	3.62 (1.05)	3.09 (1.23)	3.03 (1.21)	2.71 (1.16)	12.469 (3, 280.709)	< 0.001	0.058 (0.022, 0.096)
EE	3.28 (1.14)	3.06 (1.12)	2.81 (1.11)	2.59 (1.15)	7.858 (3, 274.850)	< 0.001	0.044 (0.013, 0.078)
IE	3.48 (1.04)	3.36 (1.14)	2.93 (1.17)	2.77 (1.22)	10.715 (3, 278.284)	< 0.001	0.056 (0.021, 0.094)
PE	3.43 (1.19)	3.25 (1.14)	3.11 (1.31)	2.84 (1.19)	4.906 (3, 276.590)	0.002	0.026 (0.003, 0.054)

Year 1: *n* = 101; Year 2: *n* = 157; Year 3: *n* = 158; Year 4: *n* = 118. CI, confidence interval. Games–Howell *post hoc* comparisons were conducted following statistically significant Welch tests.

As shown in Table 12, statistically significant differences across years of study were found in all four dimensions of classroom engagement. Skills engagement differed significantly across the four groups, Welch’s *F*(3, 280.709) = 12.469, *p* < 0.001, η^2^ = 0.058, 95% CI (0.022, 0.096). Games–Howell comparisons indicated that first-year students reported significantly higher skills engagement than second-year students, mean difference = 0.526, *p* = 0.002, 95% CI (0.155, 0.897); third-year students, mean difference = 0.583, *p* < 0.001, 95% CI (0.214, 0.951); and fourth-year students, mean difference = 0.901, *p* < 0.001, 95% CI (0.513, 1.289). No other pairwise comparisons were statistically significant.

Emotional engagement also differed significantly across years of study, Welch’s *F*(3, 274.850) = 7.858, *p* < 0.001, η^2^ = 0.044, 95% CI (0.013, 0.078). First-year students reported significantly higher emotional engagement than third-year students, mean difference = 0.469, *p* = 0.007, 95% CI (0.097, 0.842), and fourth-year students, mean difference = 0.687, *p* < 0.001, 95% CI (0.285, 1.089). Second-year students also reported higher emotional engagement than fourth-year students, mean difference = 0.471, *p* = 0.004, 95% CI (0.113, 0.830). The remaining comparisons were not statistically significant.

Interaction engagement differed significantly across the four year groups, Welch’s *F*(3, 278.284) = 10.715, *p* < 0.001, η^2^ = 0.056, 95% CI (0.021, 0.094). Games–Howell comparisons showed that first-year students reported significantly higher interaction engagement than third-year students, mean difference = 0.553, *p* < 0.001, 95% CI (0.193, 0.913), and fourth-year students, mean difference = 0.709, *p* < 0.001, 95% CI (0.313, 1.105). Second-year students also reported higher interaction engagement than third-year students, mean difference = 0.428, *p* = 0.006, 95% CI (0.091, 0.765), and fourth-year students, mean difference = 0.584, *p* < 0.001, 95% CI (0.209, 0.959).

A statistically significant difference was also observed in performance engagement, Welch’s *F*(3, 276.590) = 4.906, *p* = 0.002, η^2^ = 0.026, 95% CI (0.003, 0.054). First-year students reported significantly higher performance engagement than fourth-year students, mean difference = 0.587, *p* = 0.002, 95% CI (0.169, 1.005). Second-year students also reported higher performance engagement than fourth-year students, mean difference = 0.407, *p* = 0.024, 95% CI (0.039, 0.775). No other pairwise differences were statistically significant.

Overall, in response to RQ4b, statistically significant year-of-study differences were observed across all four dimensions of classroom engagement. First-year students generally reported the highest engagement scores, whereas fourth-year students reported the lowest. However, the patterns of significant pairwise differences varied across the four dimensions. The effect sizes were small for emotional and performance engagement and approached a moderate level for skills and interaction engagement. Thus, year of study was associated with a modest proportion of variance in classroom engagement.

The clearest year-related pattern was found in skills engagement. First-year students reported significantly higher skills engagement than students in each of the other 3 years. This finding may indicate that first-year students initially devote greater attention to developing learning strategies and adapting to university-level academic requirements. The absence of significant differences among the second-, third-, and fourth-year groups suggests that the most noticeable difference occurred between first-year students and those who had progressed beyond the initial stage of university study.

Differences in emotional and interaction engagement were more apparent between students in the earlier and later years of study. First-year students reported higher emotional and interaction engagement than third- and fourth-year students, while second-year students also reported higher scores than fourth-year students in both dimensions. This pattern may reflect the initial interest associated with entering university, as well as changes in academic demands, classroom experiences, social relationships, and learning expectations as students’ progress through their programs.

Performance engagement showed the smallest effect size. Significant differences were limited to comparisons involving fourth-year students, who reported lower performance engagement than first- and second-year students. One possible explanation is that final-year students may divide their attention among classroom learning, internships, graduation requirements, professional examinations, and employment preparation. Consequently, conventional classroom activities may become less central to their immediate academic priorities.

The observed year-of-study differences represent cross-sectional group patterns rather than within-person changes over time. Because the study compared different student cohorts at a single point in time, the results do not establish that individual students’ engagement declines as they progress through university. The observed differences may also reflect cohort characteristics, course arrangements, teaching practices, or other contextual factors.

From a practical perspective, the results suggest that support for classroom engagement should continue throughout students’ undergraduate education rather than being concentrated solely in the first year. Skills-related support may be particularly important after the transition to the second year. In the third and fourth years, educators could strengthen emotional and interaction engagement by increasing collaborative learning, providing meaningful feedback, and connecting classroom activities with students’ disciplinary interests and career goals. For final-year students, integrating classroom learning with internships, professional development, and employment preparation may help maintain the relevance of classroom participation. When implementing these forms of support, institutions may also need to consider whether courses are delivered face-to-face, online, or in hybrid formats, because comparable student satisfaction across delivery modes may coexist with different participation patterns (So et al., 2025).

## 5 Conclusion

This study examined classroom engagement among undergraduate students at a private undergraduate college in Qingdao, China. Based on the multidimensional framework proposed by Handelsman et al. (2005), classroom engagement was conceptualized in terms of skills, emotional, interaction, and performance engagement. The study investigated the levels and interrelationships of these dimensions, their contributions to the higher-order construct of classroom engagement, and their differences according to gender and year of study.

The findings supported the multidimensional structure of classroom engagement, with all four dimensions contributing substantially to the higher-order construct. Scalar measurement invariance was established across gender and year-of-study groups, and significant but modest group differences were observed. These findings extend evidence for the applicability of the four-dimensional classroom engagement framework to Chinese private higher education and demonstrate that its measurement structure is comparable across demographic groups. In addition, the results highlight the need to strengthen students’ emotional connection with classroom learning and provide sustained engagement support throughout undergraduate study, particularly during the later years. Such support should respond to students’ learning needs and classroom experiences rather than rely on fixed assumptions about gender.

Several limitations should be acknowledged. First, the participants were drawn from one private undergraduate college, limiting the generalizability of the findings to other institutions and regions. Second, the study relied on self-reported data, which may be affected by response bias. Third, the cross-sectional design does not permit causal or longitudinal interpretations; therefore, the observed year-of-study differences should not be interpreted as evidence that individual students’ engagement necessarily declines over time. Future research should include multiple institutions, employ longitudinal or mixed-method designs, and examine whether classroom climate, teacher support, learning motivation, and academic demands help explain gender- and year-related differences in classroom engagement.

## Data Availability

The raw data supporting the conclusions of this article will be made available by the authors, without undue reservation.

## Appendix

**APPENDIX 1:** Classroom Engagement Questionnaire.

No.	Dimensions	Items					
1.	Skills Engagement	Making sure to study on a regular basis. 确保定期学习。	1	2	3	4	5
2.	Skills Engagement	Putting forth effort in class. 在课堂上付出努力。	1	2	3	4	5
3.	Skills Engagement	Completing all the classroom assignments. 完成所有课堂作业。	1	2	3	4	5
4.	Skills Engagement	Being organized. 保持有条理。	1	2	3	4	5
5.	Skills Engagement	Taking good notes in class. 在课堂上做好笔记。	1	2	3	4	5
6.	Skills Engagement	Listening carefully in class. 在课堂上认真听讲。	1	2	3	4	5
7.	Skills Engagement	Coming to class every day. 每天都来上课。	1	2	3	4	5
8.	Emotional Engagement	Finding ways to make the course interesting to me. 寻找让课程变得有趣的方法。	1	2	3	4	5
9.	Emotional Engagement	Really want to learn the material. 真正想学习课程内容。	1	2	3	4	5
10.	Emotional Engagement	Finding ways to make the course material relevant to my life. 寻找使课程内容与我的生活相关的方法。					
11.	Interaction Engagement	Raising my hand in class. 在课堂上举手。	1	2	3	4	5
12.	Interaction Engagement	Asking questions when I don’t understand the instructor in class. 在课堂上当我不理解老师的讲解时提问。	1	2	3	4	5
13.	Interaction Engagement	Participating actively in small-group discussions in class. 积极参与课堂小组讨论。	1	2	3	4	5
14.	Interaction Engagement	Helping fellow students in class. 在课堂上帮助同学。	1	2	3	4	5
15.	Performance Engagement	Doing well on the tests. 在考试中表现良好。	1	2	3	4	5
16.	Performance Engagement	Being confident that I can learn and do well in the class. 有信心能够在课堂上学习并表现出色。	1	2	3	4	5
17.	Performance Engagement	Getting a good grade. 取得好成绩。	1	2	3	4	5
